# Association of Anatomical Location and Preoperative Blood Biomarkers With Arteriovenous Fistula Maturation in Hemodialysis Patients

**DOI:** 10.1155/ijvm/9737630

**Published:** 2025-09-19

**Authors:** Davood Dalil, Seyyed Mohammad Hosseini, Morteza Khavanin Zadeh

**Affiliations:** ^1^Faculty of Medicine, Shahed University, Tehran, Iran; ^2^Hasheminejad Kidney Center (HKC), Iran University of Medical Sciences (IUMS), Tehran, Iran; ^3^Advanced Diagnostic and Interventional Radiology Research Center, Tehran University of Medical Sciences, Tehran, Iran

**Keywords:** arteriovenous fistula (AVF), AVF maturation, end-stage renal disease (ESRD), hemodialysis, vascular access

## Abstract

**Introduction:** Arteriovenous fistulas (AVFs) are the best choice for providing vascular access for hemodialysis patients. AVF maturation is essential for successful hemodialysis. However, up to 60% of AVFs fail to mature due to multiple risk factors. Thus, this study is aimed at investigating the association of anatomical location and preoperative blood parameters with AVF maturity.

**Methods:** This is a retrospective cohort study of 206 patients who underwent their first AVF creation at the Hasheminejad Kidney Center, Tehran, Iran, from January 2016 to January 2019. Demographic and clinical characteristics and blood biomarkers were recorded for all patients preoperatively.

**Results:** The total maturation rate was 67.2%. The primary failure rate of AVFs was 5.8%. Regarding AVF location, wrist fistulas had a higher rate of maturation than antecubital fistulas (73.2% vs. 55.9%, *p* = 0.013, OR = 2.15). The WBC (*p* = 0.03), RBC (*p* = 0.008), and hemoglobin (*p* = 0.009) levels were lower in the matured AVFs than in the nonmatured groups. Most kidney function biomarkers were not significantly related to AVF maturation. However, the mature wrist AVFs had lower levels of albumin and calcium–phosphorus index. The wrist AVF in patients with calcium levels under 8.5 mg/dL worked more efficiently than antecubital group (76.4% vs. 52.9%, *p* = 0.015, OR = 2.87).

**Conclusion:** This study supports the evidence that wrist AVF could be a preferred AVF choice in the patients who met its clinical criteria.

## 1. Introduction

Arteriovenous fistulas (AVFs), as described by Brescia et al., are the best choice for providing efficient and preferred vascular access (VA) for hemodialysis (HD) patients due to their long-term patency and lower complication rates compared to other forms of access [1]. AVF maturation is essential for successful dialysis results. Despite much research and experience over several decades, up to 60% of AVFs fail to mature due to multiple risk factors [2]. High age, lower arm fistula, female gender, comorbidities, and vessel characteristics have been reported in studies as predictors of primary failure [3, 4].

The anatomical location, such as the upper arm or forearm, chosen to create an AVF is important to its maturation or failure. Studies showed that upper arm AVFs mature more quickly and perform better than forearm AVFs owing to the higher arterial flow velocity and larger vessel diameter [5, 6]. In addition, anatomical variation among individuals may influence the AVF maturation at certain sites. In the case of AVF failure in a specific location, the surgeon seeks to change the location of the AVF, usually from the forearm to the upper arm or vice versa. The hand of choice (right or left) for AVF placement may also change. All of these decisions would be made to provide the required VA for HD.

Preoperative blood parameters may reflect physiological conditions potentially associated with AVF outcomes. Previously, it has been found that hemoglobin (Hb) levels correlate positively with AVF maturation; higher Hb levels lead to providing adequate oxygen to the developing AVF [7]. Moreover, elevated platelet (Plt) counts have been identified during the early stages of fistula maturation, introducing the Plt as a potential biomarker for predicting AVF maturation [8]. C-reactive protein (CRP) is the other blood parameter whose potential role in predicting both early stage inflammations related to surgical trauma and late-stage stenosis associated with AVF failure has been demonstrated [9]. However, the association between AVF maturity and other blood parameters, including those related to kidney function and blood hemodynamics, has not been well investigated, and more studies are needed.

On the other hand, comparing different AVF locations in terms of blood biomarker levels during the maturation process can provide insights into their mutual influence. The anatomical location may influence the different pathophysiological and inflammatory responses, leading to differential blood biomarker expression. Therefore, this study is aimed at investigating the association of anatomical location and blood biomarkers with AVF maturity. Further, the differences between the amounts of blood parameters based on AVF location and its maturity were studied.

## 2. Materials and Methods

### 2.1. Study Design and Participants

The current study was conducted ethically in accordance with the World Medical Association Declaration of Helsinki. The Research Ethics Committee of Iran University of Medical Sciences approved all procedures performed in the current study with Approval Number: IR.IUMS.FMD.REC.1399.318. This is a retrospective observational cohort study of all patients who underwent AVF placement from January 2016 to January 2019 at the Hasheminejad Kidney Center (HKC), a tertiary care center in Tehran, Iran. Over this period, 206 ESRD patients who were referred by a nephrologist for VA underwent their first AVF creation at HKC by the same surgeon. Further, only the first fistula of each patient was analyzed during this study. Meanwhile, the exclusion criteria were as follows: (1) having no suitable vein continuity or vein diameter < 2.5 mm, (2) having no suitable artery due to atherosclerosis or artery diameter < 2.5 mm, (3) using anticoagulation drugs, and (4) having a vascular system suitable only for brachiocephalic VA, which makes a radiocephalic VA impossible. All individuals were well informed about the study and expressed their informed consent through a written form.

### 2.2. Data Collection

Demographic and clinical data, including gender, age, weight, height, body mass index (BMI), AVF location, AVF maturation, comorbidities (smoking, diabetes mellitus [DM], and hypertension [HTN]), and preoperative blood parameters including white blood cell (WBC) counts, red blood cell (RBC) counts, Hb, Plt, prothrombin time (PT), partial thromboplastin time (PTT), and international normalized ratio (INR), along with kidney function biomarkers such as blood urea nitrogen (BUN), creatinine (Cr), albumin (Alb), corrected calcium (Ca), sodium (Na), potassium (K), and phosphorus (P), were all collected. Comorbidities were defined as follows: smoking as current smoking or a history of smoking, diabetes with both insulin and noninsulin-dependent patients, and HTN or medically controlled HTN. The anatomical location of the AVF was determined by the antecubital or wrist as well as the right or left hand.

### 2.3. Evaluation of AVF Maturation

The AVF patency status was reported in three modes: primary failure, failure to maturation, and maturation, which were defined as follows: (a) Primary failure was defined as the failure of AVF in an interval from AVF creation up to 30 days thereafter. (b) Failure to maturation was defined as the AVF failing to mature between 31 and 120 days after its creation. (c) Maturation was defined as AVF patency and being efficient for dialysis after 120 days from access placement. According to the above definitions, the patients were categorized into two groups: mature AVF and immature AVF. The immature AVF group included patients with primary failure or failure to maturation of AVF.

### 2.4. Statistical Analysis

The statistical analysis was performed using SPSS software Version 27 (SPSS Inc., Chicago, Illinois, United States). The collected data were reported as mean ± standard deviation (SD) for quantitative variables and as frequency (percentage) for qualitative ones. The Kolmogorov–Smirnov test was used to analyze the normality of the data. The chi-square test, independent *t*-test (for normal distribution), Mann–Whitney *U* test (for abnormal distribution), and logistic regression analysis were used to analyze the data. A statistically significant difference was defined as a *p* value less than 0.05.

## 3. Results

**Figure 1:**
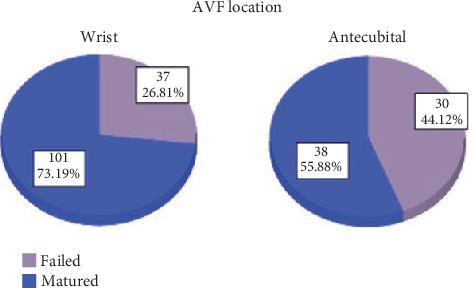
Location of AVF and maturation.

This study involved 206 patients who underwent an AVF creation for the first time (Table 1). The total maturation rate was 67.2% (*n* = 139). Among all wrist AVFs, 73.2% (*n* = 101) matured, while 55.9% (*n* = 38) of antecubital AVFs reached maturation (Figure 1). Fifty-five fistulas (26.7%) failed within 30–120 days after operation, and the remainder (*n* = 12, 5.8%) failed before 30 days postoperation. When looking at the link between the location of the AVF and the outcome (i.e., maturation or failure), it was found that wrist fistulas had a higher rate of maturation than antecubital fistulas (73.2% vs. 55.9%, *p* = 0.013, odds ratio [OR] = 2.15). Most of the AVFs were created on the left hand (89.3%), of which 68.5% were mature. Among the AVFs created on the left hand, the maturation rate of wrist fistulas was significantly more compared to antecubital ones (73.7% vs. 54.9%, *p* = 0.014).

One hundred and thirty nine patients (67.5%) were male, and 67 (32.5%) were female. Our study exhibited that the maturity of AVFs was not different between males and females (*p* > 0.05).

The mean age of participants was 53.29 ± 16.66 years; 21.4% were older than 65 years. There was a significantly higher chance of maturation for wrist fistulas compared to antecubital ones among the patients younger than 65 years old (73.2% vs. 52.0%, *p* = 0.008). However, taking all the cases into account, the mean age was not significantly different between matured and failed AVF groups (*p* = 0.828).

Of the enrolled patients, 90 were diabetic (43.7%), 155 had HTN (75.2%), and 31 were current smokers (15%) (Table 1). The present study did not find a significant relationship between the outcome of AVFs and patients' risk factors such as DM (*p* = 0.413), HTN (*p* = 0.406), and smoking (*p* = 0.386). However, the wrist AVFs had a higher maturation rate compared to antecubital AVFs among normotensive patients (77.4% vs. 40.0%, *p* = 0.007, OR = 5.15) and nonsmokers (71.7% vs. 56.5%, *p* = 0.042, OR = 1.95).

Preoperative blood parameters, including WBC, RBC, Hb, Plt, PT, PTT, and INR, and serum levels of kidney function biomarkers such as BUN, Cr, Alb, and electrolytes (i.e., Na, K, Ca, and P) of patients are described in Table 2.

The means of WBC and RBC were significantly lower in the matured AVFs than in the nonmatured groups (*p* = 0.03 and *p* = 0.008, respectively). Moreover, the mean serum level of Hb was higher in the nonmatured groups (9.56 ± 1.93 vs. 8.88 ± 1.65, *p* = 0.009). There were no significant differences between the groups regarding Plt, PT, PTT, INR, BUN, and Cr.

**Figure 2:**
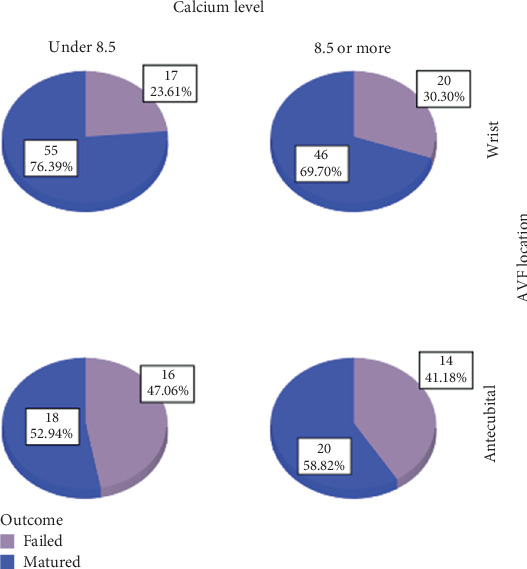
Effect of calcium level on AVF maturation in different locations.

Our results exhibited that among patients with wrist fistulas, mature AVFs had a lower mean of Alb than the nonmatured group (3.66 ± 0.54 vs. 3.51 ± 0.97, *p* = 0.032). Moreover, when patients were divided based on their serum Ca level, wrist AVFs showed better maturation rate in patients with lower Ca levels (under 8.5 mg/dL) than in patients with higher Ca levels (76.4% for wrist AVF vs. 52.9% for antecubital AVFs, *p* = 0.015, OR = 2.87) (Figure 2). Furthermore, the mean Ca–P level was compared between groups demonstrating matured AVFs on the wrist had significantly lower amounts of Ca–P compared to matured AVFs in the antecubital location (49.46 ± 14.09 vs. 58.71 ± 23.33, *p* = 0.024).

**Figure 3:**
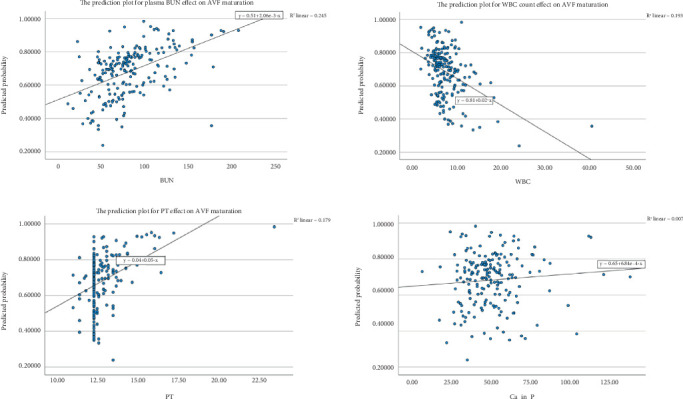
Prediction plots of AVF maturation based on logistic regression model.

A backward logistic regression analysis was conducted utilizing all independent variables to determine the most effective factors on the maturation of an AVF. The model demonstrated that the four variables—AVF location, BUN, WBC, and PT—had the strongest association with AVF maturation (Table 3). Based on our logistic regression analysis, utilizing a wrist fistula in patients can result in a 2.3-fold higher likelihood of maturation (*p* = 0.0.23, OR = 2.341, 95%CI = 1.126–4.865).  Figure 3 demonstrates the pyramid frequency plot indicating the predicted chance of an AVF maturing based on location.

**Figure 4:**
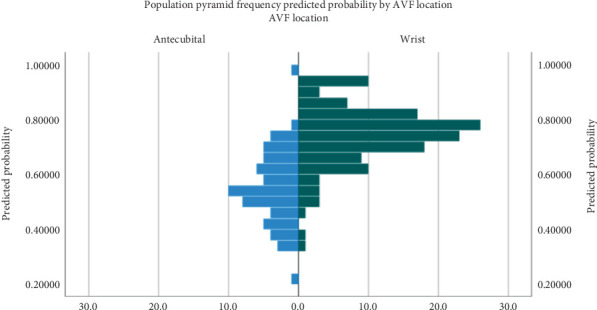
Population pyramid frequency plot for prediction of AVF maturation based on AVF location.

Furthermore, the probability of AVF maturation was much higher in people whose WBC counts were lower (*p* = 0.044, OR = 0.921, 95%CI = 0.851–0.998) and whose BUN levels were higher (*p* = 0.043, OR = 1.011, 95%CI = 1.000–1.022). The effectiveness of BUN in multivariate analysis (but not in univariate analysis) may be attributed to more multivariate models' ability to detect complex relationships, confounding factors, and variables' interactions [10].  Figure 4 shows the prediction plots for the effects of BUN, WBC, PT, and Ca–P variables on AVF maturation.

## 4. Discussion

This study investigated the effect of anatomical location and certain blood parameters, especially those related to kidney function and blood characteristics, on the maturation of AVF in HD patients. Among all the 206 AVFs created in this study, the maturity rate was 67.2%. Similarly, Huber et al., in a study of 602 participants, reported a maturation rate of 67% [11]. The overall maturation rate of AVFs in a cohort study of 300 patients was reported at 65% [12]. A meta-analysis of 318 studies, including 62,712 AVFs, reported a primary unassisted patency rate of 64% [13]. Primary failure of AVFs in our study was 5.8%, lower than that reported as 10% in a study of 831 patients [14].

The optimal AVF for permanent kidney disease (HD) is the autogenous radiocephalic wrist AVF (RCW-AVF) in the nondominant hand, which preserves upper arm vessels, shows better function, has lower rates of infection, and fewer long-term complications, but has a high rate of nonmaturation in chronic HD patients [15–17].

Our results showed that AVFs created in the wrist had a significantly higher maturation rate compared to the antecubital fistulas. Although left-hand AVFs showed higher maturation in this study, the difference was not significant (68.5% vs. 50.0%, *p* = 0.186). There are several studies that investigated the various factors affecting the maturation of AVFs. The location has always been an important factor; however, the previous studies reported some contradicting results regarding the optimal AVF location. Farrington et al. looked at 300 ESRD patients who had a new AVF created and found that the location of the AVF (forearm or upper arm) did not affect how well it developed [12]. A 4-month follow-up study of 265 patients showed that wrist AVFs had a higher maturation rate than antecubital AVFs, though not statistically significant [18]. Among 311 successful AVFs, Kaygin et al. revealed that the left radiocephalic was the most common opening site for mature AVFs [19]. Conversely, Bashar et al. reported the maturation rate of wrist and forearm AVFs at 53.5% versus 61.9%, respectively, not finding any statistical impact of AVF location on its maturation. Monroy-Cuadros et al. found that forearm AVF was associated with primary failure of fistula maturation. Moreover, they showed that left-hand AVFs had a higher patency rate [14]. These contradicting findings highlight the significance of additional variables influencing the AVF maturation.

Anastomosis angle is another significant factor that may lead to AVF failure, particularly in wrist AVFs. It affects the wall shear stress (WSS) in the outflow veins, which can lead to thrombogenesis or atherosclerosis and stenosis, resulting in AVF failure. Lee et al. found that an obtuse angle (135°) in a RCW-AVF had a higher patency rate due to lower WSS [20].

Different surgeons may have varied practices that could lead to different outcomes. A high level of surgical expertise is the other important factor that is essential for creating fistulas, particularly forearm and wrist AVFs. This is due to the small diameter of the vessels and the need to use loupe magnification in microsurgery. Regus et al. revealed that surgeon experience significantly affected the immediate and follow-up outcomes post-AVF creation for the AVF located on the forearm compared to those on the upper arm [21]. The fistulas in the present study were created by a single well-experienced surgeon, and this may be a significant factor contributing to the high maturation rate in our wrist AVFs.

Although our data indicate higher maturation rates for wrist AVFs, this is contrary to some literature that reported better results using antecubital or upper arm AVFs. It should be noted that in the present study, all patients were treated by a single experienced surgeon in a high-volume institution and this also might be a reason for the low failure rate in our cohort. Moreover, the patients qualifying for the wrist AVF according to our inclusion criteria could also be treated with the antecubital AVF, and thus, this selection may have enriched the wrist AVF group with patients of more favorable baseline vascular characteristics.

In the current study, we also found that the mature AVF group has a lower mean of WBC, RBC, and Hb levels compared to the nonmature group. Park et al. argued that a Hb level of 10 g/dL or greater may be associated with a higher chance of AVF maturation [22]. Additionally, Rodrigues et al. favored a Hb level of 10–12 g/dL for AVF maturation [23]. Khavaninzadeh et al. found that a Hb level below 8 g/dL can significantly reduce the likelihood of AVF maturation. They also observed no significant differences in AVF maturation rates among patients with Hb levels of 8–10, 10–12, or above 12 g/dL [7]. Although in the present study, the group with matured AVFs had a lower Hb level compared to the failed group, it is noteworthy that the mean of both groups was approximately in the 8–10 g/dL range.

Further, Zhang et al. and Zhu et al. showed that the WBC count might not be very different between groups with a patent AVF and those with a dysfunctional AVF. The WBC count in both groups was less than 7 × 109/L in their studies [24, 25]. Yap et al. found that the nonearly AVF failure group had a lower mean WBC count compared to the early AVF failure group. However, the difference was not statistically significant [26]. Our study found a similar and significant difference regarding WBC count between the two groups. Nevertheless, more studies should be carried out for proper elucidation of this subject.

The role of inflammation in the failure of AVF to achieve maturation has been well established. However, the exact etiology of this event is not completely recognized. The association of CRP and the erythrocyte sedimentation rate (ESR) with AVF dysfunction has previously been investigated [18, 19]. Besides, Alb is a negative phase reactant protein that is reduced in inflammation. The association between hypoalbuminemia and AVF maturation outcomes has been investigated in a few studies before [27]. Kaygin et al. showed that the serum level of Alb is significantly lower in patients with unsuccessful AVF compared with the mature AVF group [19]. In contrast, Miller et al. revealed that Alb concentration was not a remarkable predictor of AVF adequacy [28]. A meta-analysis by Zhang et al. reported no association between Alb and the risk of AVF thrombus [29]. This study similarly found no significant difference in the serum level of Alb between mature and nonmature AVFs among all patients. However, patients with matured wrist AVFs had lower levels of Alb compared to failed wrist AVFs.

One of the major causes of AVF failure is the development of venous stenosis. The distribution of mineral compounds in the blood and their precipitation on vessels may be an underlying reason for stenosis. Moreover, it has been hypothesized that the disturbance of mineral metabolism as a result of CKD may frustrate the vascular remodeling process required for AVF maturation. Previously, Olsson et al. compared the stenotic AVF veins with nonstenotic ones and found a specific deposition of Ca and P in stenotic areas, which was different from Ca compounds in atherosclerotic vessels [30]. Tüysüz and Dedemoğlu showed that high Ca–phosphate levels cause arterial stiffness, decreasing the quality of AVF and increasing the risk of AVF reoperation [31]. On the other hand, Kubiak et al. revealed that serum concentrations of Ca and P are not significantly related to the outcomes of AVF maturation by conducting a multicenter prospective cohort study of 562 patients [32]. Similarly, our study demonstrated that the serum levels of Ca and P are not associated with AVF maturation. However, we found that the mean of Ca–P levels was significantly lower in wrist AVFs than in antecubital ones among the mature AVF group, indicating the different deposition patterns of Ca and P compounds based on the structural characteristics of vessels. This may be another potential factor contributing to the higher maturation rate of wrist AVFs in our cohort.

While we demonstrated some correlations between specific blood measures and AVF maturation, we do not suggest that from our study, predictive biomarkers can be identified. Prospective ROC curve analysis and validation cohorts are needed to confirm true biomarker utility [33]. While these associations may not impact individual decision-making for AVF construction, they provide physiological footprints worth additional investigation. Blood parameters could serve as indicators for hemodynamic, inflammation, and metabolism conditions that may affect AVF performance. The correlation of these laboratory results with vascular imaging and clinical scoring systems might ultimately improve preoperative risk stratification and patient-specific planning of VA creation. Further, larger prospective studies with an incorporated prediction model are required to confirm these findings and to use them in routine clinical care.

The current study had some limitations, such as uneven patient groups and no significant differences in demographic or clinical variables. The lack of inflammatory biomarkers, medications, and the anastomosis angle evaluation may be other limitations. However, it included a large number of patients referred to a tertiary center, which could represent ESRD nationwide. Moreover, the same surgeon performing all the procedures is another strength of this study.

## 5. Conclusion

This study showed that the wrist AVFs had a higher maturation rate than antecubital AVFs, suggesting that wrist AVF could be a reasonable AVF choice in the patients who met its clinical criteria. Regarding blood parameters, we found that lower WBC, RBC, and Hb levels were associated with the maturation of AVFs. Further, we found that kidney function biomarker levels including BUN, Cr, Alb, Na, K, Ca, and P were not significantly related to AVF maturation. However, the mature wrist AVFs had lower levels of Alb and Ca–P index. Although this study advanced the knowledge of AVF maturation based on fistula location and biomarkers, future longitudinal, multicenter, and large population studies should be performed to confirm the definitive role of these factors on AVF maturation, leading to the suggestion of a comprehensive preoperative protocol to evaluate the outcome of AVF.

## Figures and Tables

**Table 1:** Comparison of AVF maturation based on its location and baseline characteristics of the study population.

	**Frequency, ** **N** **(%)**	**Maturation (%)**			**p** **value**
		**Total**	**Wrist**	**Antecubital**	
All	206 (100%)	139 (67.5%)	101 of 138 (73.2%)	38 of 68 (55.9%)	**0.013**
Gender					
Female	67 (32.5%)	44 (65.7%)	30 of 40 (75.0%)	14 of 27 (51.9%)	0.050
Male	139 (67.5%)	95 (68.3%)	71 of 98 (72.4%)	24 of 41 (58.5%)	0.108
*p* value		0.701	0.759	0.587	
Age					
≤ 65	162 (78.6%)	108 (66.7%)	82 of 112 (73.2%)	26 of 50 (52.0%)	**0.008**
> 65	44 (21.4%)	31 (70.5%)	19 of 26 (73.1%)	12 of 18 (66.7%)	0.647
*p* value		0.634	0.989	0.283	
Hand					
Left	184 (89.3%)	126 (68.5%)	98 of 133 (73.7%)	28 of 51 (54.9%)	**0.014**
Right	12 (5.8%)	6 (50.0%)	3 of 5 (60.0%)	3 of 7 (42.9%)	0.558
*p* value		0.186	0.498	0.549	
HTN					
Yes	155 (75.2%)	107 (69.0%)	77 of 107 (72.0%)	30 of 48 (62.5%)	0.239
No	51 (24.8%)	32 (62.7%)	24 of 31 (77.4%)	8 of 20 (40.0%)	**0.007**
*p* value		0.406	0.546	0.089	
DM					
Yes	90 (43.7%)	58 (64.4%)	43 of 61 (70.5%)	15 of 29 (51.7%)	0.082
No	116 (56.3%)	81 (69.8%)	58 of 77 (75.3%)	23 of 39 (59.0%)	0.070
*p* value		0.413	0.524	0.552	
Smoking					
Yes	31 (15.0%)	23 (74.2%)	20 of 25 (80.0%)	3 of 6 (50.0%)	0.132
No	175 (85.0%)	116 (66.3%)	81 of 113 (71.7%)	35 of 62 (56.5%)	**0.042**
*p* value		0.386	0.396	0.761	

*Note:* Bold values indicate statistically significant results (*p* < 0.05).

**Table 2:** Comparison of preoperative laboratory parameters based on AVF maturation and location.

	**AVF**	**Total**	**Wrist**	**Antecubital**	**p** **value**
Age	Nonmatured	52.67 ± 16.26	54.43 ± 15.40	50.50 ± 17.26	0.329
	Matured	53.58 ± 16.91	52.09 ± 16.64	57.55 ± 17.19	0.090
	*p* value	0.828	0.371	0.098	

BMI	Nonmatured	24.59 ± 4.55	24.35 ± 3.62	24.87 ± 5.54	0.660
	Matured	24.99 ± 4.33	24.67 ± 4.18	25.77 ± 4.65	0.220
	*p* value	0.545	0.684	0.469	

WBC	Nonmatured	8.56 ± 3.91	8.49 ± 3.84	8.64 ± 4.05	0.905
	Matured	7.48 ± 3.89	7.54 ± 4.38	7.30 ± 2.10	0.426
	*p* value	**0.030**	0.080	0.307	

RBC	Nonmatured	3.57 ± 0.74	3.55 ± 0.73	3.59 ± 0.75	0.796
	Matured	3.30 ± 0.62	3.26 ± 0.62	3.41 ± 0.62	0.288
	*p* value	**0.008**	**0.044**	0.273	

Hb	Nonmatured	9.56 ± 1.93	9.62 ± 2.09	9.49 ± 1.74	0.778
	Matured	8.88 ± 1.65	8.72 ± 1.64	9.29 ± 1.62	0.067
	*p* value	**0.009**	**0.009**	0.640	

Plt	Nonmatured	213.31 ± 92.89	211.49 ± 91.98	215.57 ± 95.52	0.955
	Matured	206.97 ± 80.14	206.16 ± 79.02	209.13 ± 84.08	0.966
	*p* value	0.951	0.964	0.819	

PT	Nonmatured	12.59 ± 0.73	12.55 ± 0.80	12.64 ± 0.64	0.557
	Matured	12.96 ± 1.41	12.93 ± 1.11	13.03 ± 2.08	0.356
	*p* value	0.093	0.075	0.883	

PTT	Nonmatured	29.83 ± 7.87	28.85 ± 3.37	31.01 ± 11.08	0.346
	Matured	32.08 ± 15.04	31.17 ± 11.76	34.73 ± 22.06	0.690
	*p* value	0.286	0.143	0.944	

INR	Nonmatured	1.05 ± 0.10	1.05 ± 0.11	1.04 ± 0.10	0.468
	Matured	1.10 ± 0.25	1.09 ± 0.18	1.12 ± 0.39	0.350
	*p* value	0.419	0.626	0.762	

BUN	Nonmatured	76.99 ± 32.54	74.27 ± 30.85	80.33 ± 34.75	0.762
	Matured	85.33 ± 35.59	87.24 ± 36.96	80.26 ± 31.55	0.499
	*p* value	0.138	0.156	0.674	

Cr	Nonmatured	7.90 ± 4.66	8.59 ± 5.33	7.06 ± 3.59	0.113
	Matured	8.89 ± 5.00	8.92 ± 4.79	8.82 ± 5.58	0.750
	*p* value	0.228	0.887	0.152	

Alb	Nonmatured	3.58 ± 0.52	3.66 ± 0.54	3.47 ± 0.48	0.100
	Matured	3.54 ± 0.92	3.51 ± 0.97	3.61 ± 0.77	0.161
	*p* value	0.199	**0.032**	0.431	

Ca	Nonmatured	8.30 ± 1.23	8.48 ± 1.33	8.06 ± 1.07	0.150
	Matured	8.30 ± 0.99	8.27 ± 0.98	8.38 ± 1.02	0.382
	*p* value	0.849	0.216	0.237	

P	Nonmatured	5.96 ± 2.42	5.80 ± 2.53	6.18 ± 2.29	0.385
	Matured	6.38 ± 2.42	6.09 ± 1.98	7.17 ± 3.22	0.090
	*p* value	0.293	0.355	0.295	

Ca–P	Nonmatured	48.64 ± 18.59	48.48 ± 20.20	48.85 ± 16.55	0.771
	Matured	51.95 ± 17.48	49.46 ± 14.09	58.71 ± 23.33	**0.024**
	*p* value	0.172	0.448	0.108	

Na	Nonmatured	139.31 ± 4.91	139.22 ± 5.37	139.43 ± 4.36	0.950
	Matured	138.94 ± 5.64	138.71 ± 6.14	139.53 ± 4.03	0.958
	*p* value	0.776	0.942	0.751	

K	Nonmatured	4.70 ± 0.90	4.71 ± 0.96	4.68 ± 0.84	0.879
	Matured	4.73 ± 0.91	4.66 ± 0.85	4.89 ± 1.06	0.283
	*p* value	0.789	0.568	0.578	

*Note:* Bold values indicate statistically significant results (*p* < 0.05).

**Table 3:** Logistic regression model to predict AVF maturation.

**Variables**	**p** **value**	**OR**	**95% CI for OR**	
			**Lower**	**Upper**
Wrist AVF	0.023	2.341	1.126	4.865
BUN	0.043	1.011	1.000	1.022
WBC	0.044	0.921	0.851	0.998
PT	0.070	1.435	0.971	2.120

## Data Availability

The data that support the findings of this study are available on request from the corresponding author.
